# Resilient Older Subjects with Heterozygous Familial Hypercholesterolemia, Baseline Differences and Associated Factors

**DOI:** 10.3390/ijms25094831

**Published:** 2024-04-29

**Authors:** Elisenda Climent, Antón González-Guerrero, Victoria Marco-Benedí, María-del-Mar García-Andreu, Juan Diego Mediavilla-García, Manuel Suárez-Tembra, David Benaiges, Xavier Pintó, Juan Pedro-Botet

**Affiliations:** 1 Lipid and Vascular Risk Unit, Department of Endocrinology and Nutrition, Hospital del Mar, 08003 Barcelona, Spainjpedrobotet@psmar.cat (J.P.-B.); 2 School of Medicine, Universitat Pompeu Fabra, 08003 Barcelona, Spain; anton.gonzalez01@estudiant.upf.edu; 3 Hospital Universitario Miguel Servet, IIS Aragón, CIBERCV, Universidad de Zaragoza, 50009 Zaragoza, Spain; vmarcob@iisaragon.es; 4 Hospital Ernest Lluch Martin, 50300 Calatayud, Spain; mariadelmargarciaandreu@gmail.com; 5 Vascular Risk Unit, Hospital Universitario Virgen de las Nieves, 18014 Granada, Spain; juand.mediavilla.sspa@juntadeandalucia.es; 6 Lipid and Cardiovascular Risk Unit, Hospital San Rafael, 15006 A Coruña, Spain; msuareztembra@gmail.com; 7 Lipid and Cardiovascular Risk Unit, Department of Internal Medicine, Hospital Universitario de Bellvitge, 08907 Barcelona, Spain; xpinto@bellvitgehospital.cat

**Keywords:** cardiovascular disease, heterozygous familial hypercholesterolemia, high-density lipoprotein cholesterol, lipoprotein(a), older, resilient

## Abstract

Despite elevated low-density lipoprotein (LDL) cholesterol levels, some older subjects with heterozygous familial hypercholesterolemia (HeFH) do not develop atherosclerotic cardiovascular disease (ACVD) during their lifetime. The factors related to this resilient state have not been fully established. The aim of this study was to evaluate differential characteristics between older HeFH subjects with and without ACVD and factors associated with the presence of ACVD. Subjects were part of the Spanish Atherosclerosis Society Dyslipidemia Registry, and those ≥ 70 years old and with HeFH were included. Baseline characteristics of these subjects with and without ACVD were compared. A multivariate analysis was performed to assess factors associated with the presence of ACVD. A total of 2148 subjects with HeFH were included. Resilient subjects were mostly female, younger and presented fewer comorbidities with respect to the ACVD group. Subjects without ACVD had higher baseline high-density lipoprotein (HDL) cholesterol (55.8 ± 17.1 vs. 47.9 ± 15.4 mg/dL; *p* < 0.001) and lower lipoprotein(a) [Lp(a)] (53.4 ± 67.9 vs. 66.6 ± 85.6 mg/dL; *p* < 0.001) levels with respect to those in the ACVD group. Lp(a) and the presence of ≥3 risk factors were associated with the presence of ACVD.

## 1. Introduction

Heterozygous familial hypercholesterolemia (HeFH), the most frequent human metabolism monogenic disorder, is characterized by elevated plasma low-density lipoprotein (LDL) cholesterol levels due to mutations in the genes encoding for the LDL receptor [1], apolipoprotein (Apo) B [2], proprotein convertase subtilisin/kexin-type 9 (PCSK9) [3] or apo E [4]. Subjects with this familial condition have a raised risk of atherosclerotic cardiovascular disease (ACVD) [5]. If LDL cholesterol goals are not reached, patients under the age of 40 years with HeFH present a 100-fold increased risk of coronary heart disease mortality. Moreover, around 50% of HeFH subjects will survive to 60 years, with only 20% reaching 70 years of age [6]. 

Although ACVD in HeFH is largely driven by LDL cholesterol concentrations, its prevalence in HeFH is extremely variable, even in subjects sharing the same pathogenic mutation [7]. Thus, it has been speculated that other factors could also play a role in the inter-individual ACVD variation in this specific population [8]. In this respect, it has also been pointed out that a subgroup of HeFH subjects of a more advanced age do not develop ACVD during their lifetime [9,10]. However, the possible associated factors to the cardiovascular-event free survival in these individuals have not been fully established. In this regard, a recent study [11] with HeFH subjects aged ≥ 65 years, observed that factors such as age, gender, carrying a defective mutation, presence of comorbidities and lipid subfractions were related to the presence or not of ACVD. However, further evidence on whether this resilience state is maintained over time, or if these same factors apply for resilient HeFH subjects of a more advanced age, is, at present, very scarce and inconsistent. 

Thus, the aim of the present study was to evaluate the differential baseline characteristics between older HeFH subjects ≥ 70 years old with and without established ACVD. Moreover, possible factors associated with the presence of cardiovascular events in these HeFH subjects were also evaluated.

## 2. Results

### 2.1. Baseline Characteristics

A total of 2148 subjects with HeFH were included in the study (1583 without- and 565 with ACVD). The subjects with no ACVD were mostly women (63% vs. 31%; *p* < 0.001) compared with the ACVD group. Moreover, patients in the ACVD-naive group were younger (76.6 ± 5.9 vs. 77.9 ± 6.6 years; *p* < 0.001) and presented a lower BMI (27.8 ± 4.2 vs. 28.8 ± 4.3 kg/m^2^; *p* < 0.001) in comparison to those with ACVD. 

As to family history, the percentage of premature ACVD in a first-degree relative was higher in those with ACVD diagnosis with respect to the resilient group (25.3% vs. 19.7%; *p* < 0.001). 

Regarding personal history, the presence of other comorbidities, such as T2DM and hypertension, was also higher in those with ACVD (42.1% vs. 25.3 and 53.8% vs. 44.5%, respectively; *p* < 0.001). Finally, a greater percentage of the HeFH subjects englobed in the ACVD group had a positive genetic mutation (23.4% vs. 18.1%; *p* < 0.001). The rest of the baseline characteristics used to compare those subjects with and without ACVD are detailed in Table 1. 

Regarding baseline lipid profiles, HeFH subjects without ACVD had higher total (299.4 ± 89.6 vs. 284.9 ± 112.8 mg/dL; *p* = 0.003) and HDL (55.8 ± 17.1 vs. 47.9 ± 15.4 mg/dL; *p* < 0.001) cholesterol levels with respect to those in the ACVD group. However, baseline Lp(a) levels (66.6 ± 85.6 vs. 53.4 ± 67.9 mg/dL; *p* < 0.001) were higher in the ACVD group. The remaining baseline characteristics of lipid subfractions are described in Table 2.

### 2.2. Current Lipid Profile

During follow-up, those in the non-ACVD group presented higher total (198.9 ± 52.2 vs. 172.0 ± 51.3 mg/dL; *p* < 0.001) and LDL (126.3 ± 48.4 vs. 96.0 ± 49.3 mg/dL; *p* < 0.001) cholesterol levels. HDL cholesterol levels were also higher in the resilient group (56.7 ± 16.0 vs. 48.6 ± 14.7 mg/dL; *p* < 0.001). The rest of current lipid-subfractions levels are presented in Table 3.

### 2.3. Factors Associated with the Presence of ACVD

A binary logistic regression analysis was realized to assess factors related to the presence of ACVD in HeFH subjects. In this respect, Lp(a) (OR 1.002, 95% CI: 1.000–1.004; *p* = 0.019) and the presence of ≥3 cardiovascular risk factors (OR 2.795, 95% CI: 1.950–4.005; *p* < 0.001) were associated with the presence of ACVD. The rest of the results regarding the regression analyses are further detailed in Table 4.

## 3. Discussion

The present study included a total of 2148 older HeFH subjects, with almost 70% of them presenting without ACVD. HeFH patients free of cardiovascular events were younger and mostly female, and a small percentage presented comorbidities (such as T2DM, and hypertension) and a confirmed positive genetic mutation in canonical FH genes. As to baseline lipid profile, HDL cholesterol levels were higher and Lp(a) levels were lower in the non-ACVD group. During follow-up, those HeFH subjects free of ACVD events presented higher total, HDL, non-HDL and LDL cholesterol levels, with lower triglyceride levels as to the ACVD group. Finally, the regression analysis found Lp(a) concentrations and the presence of ≥3 cardiovascular risk factors as the independent predictive factors of the presence of ACVD in this older HeFH population.

HeFH subjects present elevated LDL cholesterol levels, and this in turn leads to an early development of atherosclerosis, causing both peripheral and coronary artery disease [12]. Moreover, receiving lipid-lowering specifically aimed to achieve LDL cholesterol goals can effectively decrease cardiovascular event rates. However, it has been speculated that a subgroup of these HeFH subjects may be naturally protected from suffering ACVD [13,14]. 

As previously detailed, resilient HeFH subjects of the present study were younger, mostly female, presented fewer comorbidities, and had higher HDL and lower Lp(a) concentrations at baseline in comparison to the ACVD group. These results fully agree with those observed in the SAFEHEART study in genetically defined patients ≥ 65 years old with HeFH diagnosis in Spain [11]. In that study [11], the resilient group profile also englobed those subjects with a younger age, female gender, absence of hypertension, elevated HDL cholesterol and decreased Lp(a) levels. However, it must be acknowledged that these patients were younger in comparison to our cohort, which consisted of people all ≥ 70 years of age. Similarly to our study, Khoury et al. [15] also evaluated cardioprotective markers among HeFH subjects ≥ 70 years, confirming once again the relationship between the female sex and high HDL cholesterol in reducing FH-related ACVD risk. They also observed high adiponectin levels and a non-smoking habit as other significant contributory factors. 

Thus, it seems clear that specific factors such as age, gender, absence of comorbidities or certain lipid subfractions are tightly related to the presence or not of ACVD in HeFH subjects. First of all, a younger age implies a lower increased LDL cholesterol exposure burden and could explain its relationship with a more resilient profile. However, in the present study we are evaluating the ACVD-free evolution in the older population, hence we could speculate that other protective factors aside from age must also be involved in decreasing cardiovascular risk in these subjects.

Focusing on gender, previous epidemiological studies already observed sex differences in ACVD incidence, emphasizing that cardiovascular events usually appear up to 10 years later in women in comparison to men. A possible explanation for this protective effect in the female gender is the endogenous exposure to estrogen, as it has been described that menopause is associated with an increased risk of coronary heart disease [16]. However, once again, other unknown factors may also be involved, as we have observed that the protective effect of the female gender is still present in more advanced age groups (beyond 70 years of age) despite already having achieved post-menopausal status [17].

Furthermore, other risk factors, such as hypertension and T2DM, increase the cardiovascular risk. Thus, it seems plausible that a lower proportion of resilient older HeFH subjects presented associated comorbidities in comparison to those with previous ACVD [18]. In this line, the multivariate analysis found that presenting at least three cardiovascular risk factors (including male sex, hypertension, T2DM, obesity and smoking habit) was tightly associated with the presence of ACVD.

Moving forward to lipid profile and the different subfractions, both the present study and previous publications [15,19] concur in the protective role of high HDL cholesterol levels as to ACVD prevention. However, the underlying mechanisms behind HDL’s protective role in atherogenesis remain undefined. To shed light on this matter, Melnes et al. [20] analyzed the lipoprotein profile of those subjects free of cardiovascular events. In this study, the resilient older HeFH subjects had significantly higher levels of large and extra-large HDL cholesterol particles compared to the ACVD group. Hence, these results introduce the idea that not only the number but also the composition, size and functionality of HDL particles play a role in their atheroprotective effect [21,22].

ACVD-free HeFH subjects also presented lower baseline Lp(a) levels in comparison to the ACVD group, and Lp(a) was found to be one of the predictive factors of cardiovascular disease. Previous studies had also noted an association between Lp(a) levels and the presence of cardiovascular events in FH subjects, regardless of the presence of other cardiovascular risk factors [11,23,24,25].

Finally, we believe the results obtained in the present study, together with previous data are of great clinical interest. Thus, knowing the factors related to the absence of ACVD in the HeFH population, or, on the contrary, identifying those non-resilient subjects, may guide on which subjects need a prompter and a more aggressive lipid-lowering strategy [26]. Moreover, a deeper understanding of the metabolic profile in event-free older HeFH may help develop novel preventive lipid-lowering therapies. Although resilient subjects are less likely to present ACVD, we must not forget that a specific LDL cholesterol goal must be reached in these HeFH subjects. As observed in the present study, during follow-up, those cardiovascular event-free subjects, although presenting higher HDL cholesterol levels, also presented more elevated LDL cholesterol levels and did not reach current recommendation guidelines [27]. We could speculate that these may be explained, at least in part, by an “infra-treatment” in those HeFH subjects without previous ACVD diagnosis. In this regard, Schreuder et al. [28] have very recently analyzed the attainment of LDL cholesterol target levels and the reasons for not reaching these in 3178 HeFH subjects. They noted that only 26.9% of women and 28.9% of men reached LDL cholesterol goals, this percentage decreasing to 17.2% and 25.8% in ACVD-diagnosed women and men, respectively. Not surprisingly, the reasons observed for not accomplishing LDL cholesterol targets were insufficient effect of maximum lipid-lowering therapy, as well as with the presence of side effects.

The present study had some limitations. First, the presence or not of ACVD was extracted from the patients’ medical records, resulting in a possible associated lack of information in some of the cases. Second, all subjects included were extracted from the Dyslipidemia Registry of the Spanish Arteriosclerosis Society. These patients are therefore treated and followed at specialized lipid clinics and, consequently, the present results cannot be extrapolated to the whole population. Moreover, data on diet and physical activity, which play a role in cardiovascular risk, were not available for all subjects. Finally, the design of the study was observational, and lipid-lowering treatment was assigned to each patient following clinical criteria but not a standardized protocol.

## 4. Materials and Methods

### 4.1. Study Characteristics

Subjects included in the present study were part of the Spanish Atherosclerosis Society (SEA) Dyslipidemia Registry. The inclusion criteria were patients ≥ 70 years old regardless of gender, clinically diagnosis of HeFH, active follow-up in the Lipid Unit of their reference hospital, blood analysis including complete lipid profile [total, LDL, high-density lipoprotein (HDL) cholesterol and triglycerides] in the last 6 months, principal investigator participating in their clinical follow-up and accessibility to medical record, and previous signature of informed consent. Exclusion criteria were patients who did not meet all the inclusion criteria or lacked a signature of informed consent. 

### 4.2. Study Variables

The following parameters were collected for all patients, including age and gender, history of diabetes and hypertension, as well as other risk factors such as smoking status or the presence of overweight or obesity. Moreover, a complete lipid profile (including total cholesterol, LDL cholesterol, HDL cholesterol, triglyceride and non-HDL cholesterol concentrations) at baseline and follow-up was also registered, as well as lipoprotein(a) [Lp(a)] levels and lipid-lowering therapy. Finally, the presence of ACVD and age at diagnosis were also recorded. 

### 4.3. SEA Dyslipidemia Registry

The SEA Dyslipidemia Registry was created in 2013 as an active online registry in which 65 certified lipid clinics across Spain could report cases of various types of primary hyperlipidemias [29]]. Anonymous clinical data collection in this registry was approved by a central ethics committee (Comité Ético de Investigación Clínica de Aragón, Zaragoza, Spain, approval code: No. 15/2018, approval date: 28 June 2018), and participants gave their written informed consent. Minimum data for the inclusion of cases in the registry include age, sex, smoking status, history of diabetes, hypertension, presence of ACVD and age at diagnosis, body mass index (BMI), waist circumference, and complete lipid profile without lipid-lowering treatment at diagnosis. HeFH was defined according to Dutch Lipid Clinic Network (DLCN) scoring. Subjects with scores ≥ 3 points (definite, probable or possible) were included in this analysis.

The registry is designed so that, at least once a year, the data on the clinical evolution of the included patients is updated with new anthropometric data, changes in risk factors or medications, and the appearance of new cardiovascular events. ACVD is defined as coronary heart disease (myocardial infarction, acute coronary syndrome with stenosis > 50% of a main coronary artery and coronary revascularization), stroke (ischemic and hemorrhagic), aortic aneurysm and lower limb ischemia (intermittent claudication with ankle/brachial index < 0.9 or revascularization of lower limb arteries). Arterial hypertension is defined as systolic blood pressure (SBP) ≥ 140 mm Hg or diastolic blood pressure (DBP) ≥ 90 mm Hg or self-reported use of antihypertensive medication. Type 2 diabetes mellitus (T2DM) is defined as fasting blood glucose > 125 mg/dL, HbA1c ≥ 6.5% or taking blood glucose-lowering drug therapy. Current smoking is considered as current smoking or having smoked in the last year. A former smoker is defined as a subject has smoked at least 50 cigarettes in their lifetime but who has not smoked in the last year.

### 4.4. Statistical Analysis

Data were expressed as mean ± standard deviation for continuous variables and as percentages and frequencies for categoric variables. Normality of the models was evaluated visually using the Kolmogorov–Smirnov test. For skewed variables [triglycerides and Lp(a)], a logarithmic transformation was used to achieve normality. Student’s *t*-test was performed to assess differences between two means. χ^2^ or Fisher’s exact tests were used to evaluate the degree of association among categorical variables. A multivariate analysis with a step-back procedure was performed to evaluate factors independently associated with the presence of ACVD in older subjects with HeFH. A 2-sided *p*-value < 0.05 was considered statistically significant. Statistical analysis was calculated with SPSS (version 25 for Windows; SPSS, Chicago, IL, USA).

## 5. Conclusions

In the present study, older HeFH subjects free of ACVD were younger and mostly female, while a small percentage presented associated comorbidities or were under lipid-lowering treatment. Moreover, HeFH resilient older subjects presented a more favorable lipid profile at baseline with higher HDL cholesterol and lower Lp(a) levels. During follow-up, those subjects free of ACVD showed higher total, HDL, non-HDL and LDL cholesterol levels, with lower triglyceride levels in comparison to the ACVD group. Finally, Lp(a) and the presence ≥ 3 cardiovascular risk factors were found to be associated with the presence of ACVD in the older HeFH population.

## Figures and Tables

**Table 1:** Baseline characteristics of the 2148 HeFH subjects.

Variable	Non-ACVD	ACVD	*p*
Total, n	1583	565	
Male sex, n (%)	586 (37.0)	389 (68.8)	**<0.001**
Age (years), mean ± SD	76.6 ± 5.9	77.9 ± 6.6	**<0.001**
BMI (kg/m^2^), mean ± SD	27.8 ± 4.2	28.8 ± 4.3	**<0.001**
**Family History**			
Paternal ACVD, n (%)	254 (16.0)	91 (16.1)	**<0.001**
Maternal ACVD, n (%)	158 (10.0)	56 (9.9)	**<0.001**
Premature ACVD in a first-degree relative, n (%)	312 (19.7)	143 (25.3)	**<0.001**
**Personal History**			
T2DM, n (%)	400 (25.3)	238 (42.1)	**<0.001**
Hypertension, n (%)	704 (44.5)	315 (53.8)	**<0.001**
Systolic blood pressure (mmHg), mean ± SD	132.7 ± 27.6	130.2 ± 53.7	0.138
Diastolic blood pressure (mmHg), mean ± SD	78.5 ± 23.7	73.8 ± 44.7	**0.009**
Tendon xanthoma, n (%)	157 (9.9)	66 (11.7)	**<0.001**
Corneal arch, n (%)	365 (23.1)	131 (23.2)	**<0.001**
Current smokers, n (%)	193 (12.2)	55 (9.7)	**<0.001**
Positive genetic mutation, n (%)	287 (18.1)	132 (23.4)	**<0.001**
Type genetic mutation, n (%)	*LDLR* 267 (93.0) *APOB* 18 (6.3) *PCSK9* 2 (0.7) *APOE* 0 (0)	*LDLR* 121 (91.7) *APOB* 10 (7.5) *PCSK9* 0 (0) *APOE* 1 (0.8)	0.342

ACVD: atherosclerotic cardiovascular disease; BMI: body mass index; T2DM: type 2 diabetes mellitus. Bold values indicate results with statistical significance.

**Table 2:** Baseline lipid profile (without lipid-lowering treatment) of the HeFH subjects.

Variable	Non-ACVD	ACVD	*p*
Total cholesterol (mg/dL), mean ± SD	299.4 ± 89.6	284.9 ± 112.8	**0.003**
HDL cholesterol (mg/dL), mean ± SD	55.8 ± 17.1	47.9 ± 15.4	**<0.001**
Non-HDL cholesterol (mg/dL), mean ± SD	243.6 ± 87.2	237.0 ± 109.7	0.099
LDL cholesterol (mg/dL), mean ± SD	194.4 ± 98.7	187.8 ± 115.0	0.112
Triglycerides (mg/dL), mean ± SD	208.3 ± 378.5	206.9 ± 227.3	0.467
Lp(a) (mg/dL), mean ± SD	53.4 ± 67.9	66.6 ± 85.6	**<0.001**

HDL: high-density lipoprotein; LDL: low-density lipoprotein; Lp(a): lipoprotein(a). Bold values indicate results with statistical significance.

**Table 3:** Current laboratory parameters (with lipid-lowering treatment) of the HeFH subjects.

Variable	Non-ACVD	ACVD	*p*
Total cholesterol (mg/dL), mean ± SD	198.9 ± 52.2	172.0 ± 51.3	**<0.001**
HDL cholesterol (mg/dL), mean ± SD	56.7 ± 16.0	48.6 ± 14.7	**<0.001**
Non-HDL cholesterol (mg/dL), mean ± SD	142.0 ± 48.6	123.4 ± 48.6	**<0.001**
LDL cholesterol (mg/dL), mean ± SD	126.3 ± 48.4	96.0 ± 49.3	**<0.001**
Triglycerides (mg/dL), mean ± SD	144.2 ± 152.8	160.1 ± 148.1	**<0.001**

HDL: high-density lipoprotein; LDL: low-density lipoprotein. Bold values indicate results with statistical significance.

**Table 4:** Binary logistic regression analysis to assess factors related to the presence of ACVD in HeFH subjects.

	OR (95% CI)	*p*
Male sex	1.698 (0.768–3.758)	0.191
Age	1.053 (0.988–1.122)	0.115
BMI, kg/m^2^	0.995 (0.975–1.015)	0.604
T2DM	1.058 (0.456–2.455)	0.896
Hypertension	0.909 (0.410–2.017)	0.815
Active smoking	1.833 (0.403–8.340)	0.433
Total cholesterol (mg/dL)	0.962 (0.921–1.006)	0.087
HDL cholesterol (mg/dL)	1.035 (0.958–1.119)	0.383
LDL cholesterol (mg/dL)	1.030 (0.991–1.070)	0.130
Triglycerides (mg/dL)	1.012 (1.000–1.024)	0.050
Lp(a) (mg/dL)	1.002 (1.000–1.004)	**0.019**
Positive genetic mutation	1.231 (0.659–2.297)	0.515
Presence ≥ 3 CV risk factors	2.795 (1.950–4.005)	**<0.001**
Statin therapy > 30 years or initiation < 40 years of age	0.442 (0.024–8.306)	0.586
Lp(a) > 50 mg/dL	1.183 (0.099–14.172)	0.894

BMI: body mass index; CV: cardiovascular; HDL: high-density lipoprotein; HeFH: heterozygous familial hypercholesterolemia; LDL: low-density lipoprotein; Lp(a): lipoprotein(a); T2DM: type 2 diabetes mellitus. CV risk factors include male sex, hypertension, T2DM, obesity (BMI > 30 kg/m^2^) and active smoking. Bold values indicate results with statistical significance.

## Data Availability

Data is unavailable due to privacy restrictions.
